# Microenvironmentally controlled secondary structure motifs of apolipoprotein A-I derived peptides

**DOI:** 10.1007/s11010-014-2050-2

**Published:** 2014-04-20

**Authors:** Paola Mendoza-Espinosa, Danai Montalvan-Sorrosa, Victor García-González, Abel Moreno, Rolando Castillo, Jaime Mas-Oliva

**Affiliations:** 1 Instituto de Fisiología Celular, Universidad Nacional Autónoma de México, 04510 México D.F, México; 2 Instituto de Física, Universidad Nacional Autónoma de México, 04510 México D.F, México; 3 Instituto de Química, Universidad Nacional Autónoma de México, 04510 México D.F, México; 4 División de Investigación, Facultad de Medicina, Universidad Nacional Autónoma de México, 04510 México D.F, México

**Keywords:** Apolipoprotein A-I, Amyloidogenic peptides, Disorder-to-order transitions

## Abstract

The structure of apolipoprotein A-I (apoA-I), the major protein of HDL, has been extensively studied in past years. Nevertheless, its corresponding three-dimensional structure has been difficult to obtain due to the frequent conformational changes observed depending on the microenvironment. Although the function of each helical segment of this protein remains unclear, it has been observed that the apoA-I amino (N) and carboxy-end (C) domains are directly involved in receptor-recognition, processes that determine the diameter for HDL particles. In addition, it has been observed that the high structural plasticity of these segments might be related to several amyloidogenic processes. In this work, we studied a series of peptides derived from the N- and C-terminal domains representing the most hydrophobic segments of apoA-I. Measurements carried out using circular dichroism in all tested peptides evidenced that the lipid environment promotes the formation of α-helical structures, whereas an aqueous environment facilitates a strong tendency to adopt β-sheet/disordered conformations. Electron microscopy observations showed the formation of amyloid-like structures similar to those found in other well-defined amyloidogenic proteins. Interestingly, when the apoA-I peptides were incubated under conditions that promote stable globular structures, two of the peptides studied were cytotoxic to microglia and mouse macrophage cells. Our findings provide an insight into the physicochemical properties of key segments contained in apoA-I which may be implicated in disorder-to-order transitions that in turn maintain the delicate equilibrium between both, native and abnormal conformations, and therefore control its propensity to become involved in pathological processes.

## Introduction

Apolipoprotein A-I (apoA-I) is considered the major component of high-density lipoproteins (HDL) and plays a key role in reverse cholesterol transport, a process that removes excess cholesterol from the cell membranes of peripheral tissues and, therefore, works as a protection mechanism against the development of atherosclerosis [1].

Natively unfolded proteins, such as apoA-I [2], have recently garnered significant interest and led to coin the term unfoldome [3]. Disordered domains in natively unfolded proteins have been associated with protein and lipid transport, transcriptional regulation [4], and a large number of diseases including amyloidosis, cancer, diabetes, and neurodegenerative disorders [5]. Nuclear magnetic resonance (NMR) and electron paramagnetic resonance (EPR) have demonstrated that the amino (N) and carboxy-end (C) segment of lipid-free apoA-I present variable secondary structures and, therefore, considerable plasticity [6–11].

Several HDL models that go from a discoidal to a spherical shape have suggested that the exposed N- and C-terminal domains of apoA-I interact with lipids [12–15], and shown that the presence of cholesterol and phospholipids determines whether the apoA-I structure is present in an open or closed conformation [6]. The highly hydrophobic C-terminal domain anchors apoA-I to membranes [16], whereas both the N- and C-terminal domains have been shown to play roles in receptor-dependent binding and lipid remodeling of discoidal HDL particles. Antibody binding and cross-linking studies have shown that the apoA-I N- and C-terminal domains interact with the scavenger receptor B1 (SRB1), responsible for the transfer of free cholesterol and cholesteryl-esters to the liver [17, 18]. On the other hand, we have recently shown that several α-helical peptides derived from the N- and C-terminal domains of apoA-I are able to activate the plasma enzyme lecithin cholesterol acyltransferase (LCAT) [19]. While several α-helical structures of apoA-I and their interactions with proteins and lipids have been widely studied, other protein structural features of apoA-I, such as β-sheets, have not been systematically investigated. From the few studies performed, site-directed spin-labeling electron paramagnetic resonance spectroscopy (SDSL-EPR) has revealed the presence of a short β segment at both the N- and the C-terminal regions of the lipid-free form of apoA-I [7, 20].

Although several of the conformational transitions in apoA-I are prone to be dependent on their microenvironment, little is known related to its capacity to acquire, depending on this microenvironment, a secondary structure susceptible to aggregation, such as globular forms and β-sheets.

The most hydrophobic and disordered segments of the N- and C-end regions of apoA-I were identified through in silico analysis performed for the complete apoA-I sequence. The physicochemical analysis included properties such as hydrophobic moment, charge, average hydrophobicity associated to steric zippers, theoretical average velocity of aggregation, and possible patterns including residues promoting membrane interactions, self-assembling and aggregation. According to these data, we studied the structural features of short peptides derived between N- and C-terminal domains of apoA-I. We identified several apoA-I modules that promote self-assembly and aggregation, and found that the positions of specific key aromatic residues may affect lipid binding. Our results shed light on the mechanisms that regulate localized conformational transitions that in turn might affect the way apoA-I interacts with HDL particles.

## Materials and methods

### Peptide synthesis and sample preparation

Three peptides were synthesized (purity > 98 %) based on the sequence and functional information reported: apoA-I [9–24]/D^9^-RVKDLATVYVDVLKD^24^ (abbreviated in the present study as DRV^(9–24)^), apoA-I[45–63]/K^45^-LLDNWDSVTSTFSKLREQ^63^ (abbreviated in the present study as KLL^(45–63)^), and apoA-I[221–239]/V^221^-LESFKVSFLSALEEYTKK^239^ (abbreviated in the present study as VLES^(221–239)^) (GenScript Corporation Piscataway, NJ). Lyophilized peptides were dissolved at 1 mg/mL and diluted to a final concentration of 200 μg/mL in ultrapure water (18.2 MΩ·cm, pH 6.8), in the presence of 20 mM 1-lauroyl-2-hydroxy-*sn*-glycero-3-phosphocholine (Lyso-C_12_PC, CAS RN: 20559-18-6) or 40 % 2,2,2-trifluoroethanol (TFE, CAS RN: 75-89-8). Protein concentrations were determined measuring absorbance at 280 nm.

### *In silico* analysis

The primary structure of apoA-I was placed into multiple algorithms to predict disorder-susceptible regions, hydrophobic clusters and aggregation-prone regions. The PONDR-FIT algorithm, a meta-predictor that joins the results of six programs (PONDR-VLXT, PONDR-VSL2, PONDR-VL3, FoldIndex, IUPred, and TopIDP) and forms an artificial consensus from these results, was used to predict conformational disorder [21]. Hydrophobic segments were predicted using the hydrophobic cluster analysis (HCA) server [22]. Regions prone to form amyloid fibrils and globular structures were predicted using the Zyggregator server [23]. Zyggregator uses an algorithm that considers patterns of hydrophobicity, as well as the polar and aromatic amino acid content of amyloidogenic proteins. The prediction of the aggregation rate was calculated using the equation of Dubay et al. [24], considering several factors that influence aggregation, such as pH, ionic force, the presence of specific amino acid sequences, net charge, and total hydrophobicity.

### Circular dichroism spectroscopy

Far-ultraviolet (UV) circular dichroism (CD) spectra were recorded on an Aviv 62DS spectropolarimeter in a 0.1 cm quartz cell using an average time of 2.5 s and a step size of 0.5 nm over a wavelength range of 190–260 nm. Sample concentration was determined before each CD measurement and following baseline correction, ellipticity was converted to mean molar ellipticity (Θ, deg cm^2^ dmol^−1^). Secondary structure content was calculated at 190–260 nm using the circular dichroism neural network (CDNN) based software [25].

### Transmission electron microscopy and atomic force microscopy

Samples of peptide solutions in water were collected after 0, 24, 48, 72, 96 h and 120 days of incubation at 4 °C and observed using transmission electron microscopy (TEM) with a JEM-1200EX11 JEOL microscope (70 kV) and atomic force microscopy (AFM) performed with an AFM Digital Instruments/Veeco. Peptide solutions for TEM were deposited on carbon-coated transmission electron microscopy grids and stained with 2 % uranyl acetate. Aliquots of peptide solutions in water were deposited onto freshly cleaved mica, dried under laminar flow for 5 min, and visualized by AFM. Images were obtained using a Multimode microscope (Digital Instruments/Veeco) and a Nano Scope IIIa (Digital Instruments/Veeco) control system. Images (5.0 × 5.0 µm) were obtained in contact mode at a scan frequency of 2 Hz using silicon nitride (Si_3_N_4_) AFM tips.

### Viability assays and optical microscopy

RAW (mouse macrophage, ATCC CRL-2467) and EOC cells (mouse microglia, ATCC TIB-71) were grown as previously described by us [26]. Macrophage and microglial cells were placed into 96-well plates at a density of 1 × 10^4^ cells/well (100 µL/well) and incubated for 24 h at 37 °C. Serial dilutions of aged peptide solutions (120 days at 4 °C) were prepared in Opti-MEM reduced-serum medium (OptiMEM) without phenol red. Incubations were performed for 20 h, and cell viability estimated by measuring the cellular reduction of MTT (3-(4, 5-dimethylthiazol-2-yl)-2, 5-diphenyltetrazolium bromide) as previously described by us [26]. Cell images were observed by optical microscopy at 40× magnification, processed and stored as TIF using an Olympus IX71 microscope and the Image-Pro 3DS 6.0 software.

## Results

### *In silico* analysis of apoA-I and apoA-I-derived peptides

**Fig. 1:**
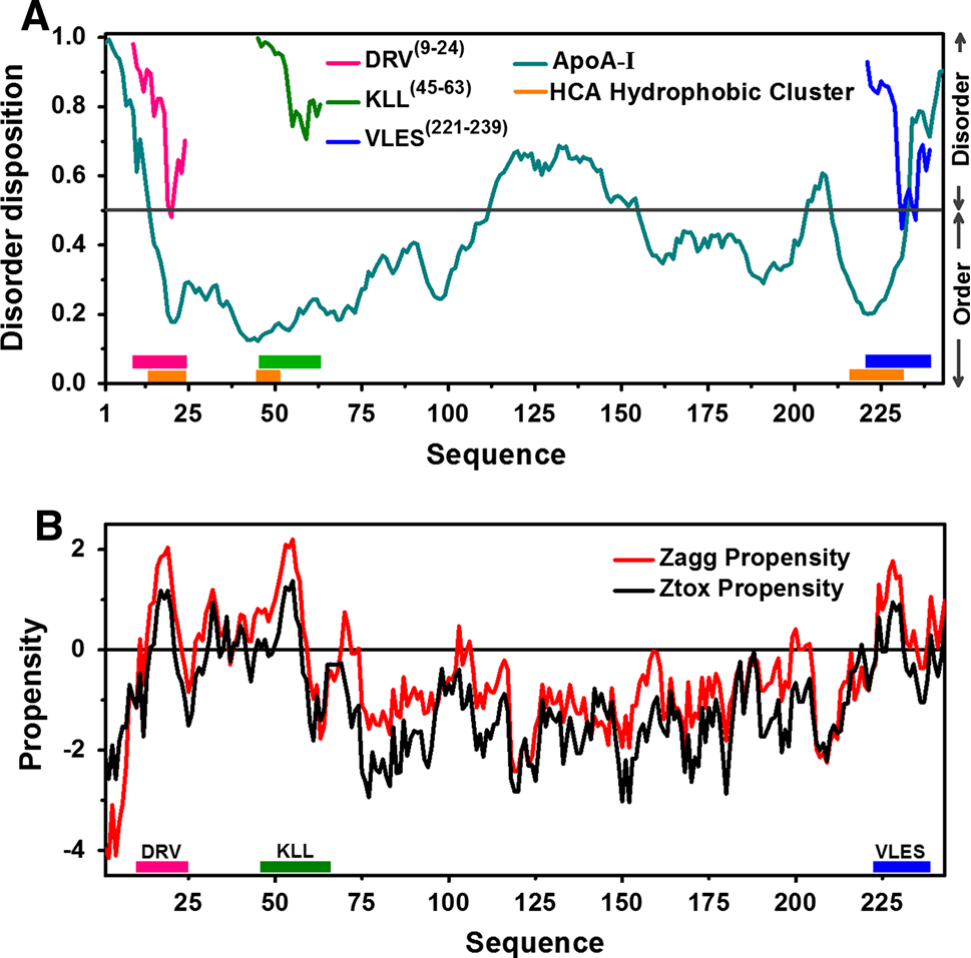
Disorder and aggregation predictions for apoA-I**. a** The disorder profiles were created with PONDR-FIT and the hydrophobic clusters predicted using the HCA server. Propensities to disorder for DRV^(9–24)^ and VLES^(221–239)^ overlapped with the apoA-I disorder profile. **b** Aggregation profiles of apoA-I were obtained using Zyggregator and prediction of propensity to generate amyloid fibrils and globular structures using Zagg propensity and Ztox propensity respectively

Using the PONDR-FIT algorithm, four disorder-prone regions (amino acids 1–13, 112–154, 204–210, 233–243) were identified in apoA-I (Fig. 1a). The disorder propensity graph shows that segments corresponding to peptides DRV^(9–24)^ and VLES^(221–239)^ present a higher tendency toward an unstructured conformation than the highly ordered segment KLL^(45–63)^. Nevertheless, when individual peptide sequences rather than the entire protein sequence were analyzed, we observed that peptide KLL^(45–63)^ presented the highest tendency to become disordered (Fig. 1a). Using the HCA server, three highly hydrophobic segments (amino acids 13–22, 45–49, 216–232) were predicted that precisely correspond to DRV^(9–24)^, KLL^(45–63)^, and VLES^(221–239)^ (Fig. 1a). In addition, Zyggregator identified several native apoA-I sites with the propensity to form amyloid fibrils and globular structures (amino acids 15–20, 50–57, and 224–230). These sites also correspond to peptides DRV^(9–24)^, KLL^(45–63)^, and VLES^(221–239)^ (Fig. 1b). The theoretical prediction for aggregation rates (Zag propensity) indicates that peptide VLES^(221–239)^ could form amyloid-like aggregates faster than the other peptides tested (Table 1).

**Table 1:** Physicochemical properties of peptides DRV^(9–24)^, KLL^(45–63)^, and VLES^(221–239)^

Peptide	DRV (9–24)	KLL (45–63)	VLES (221–239)
Amino acid sequence^a^			
Average charge	−1	0	0
Average hydrophobicity (kcal/mol)	−0.116	−0.151	0.118
Hydrophobic moment µH (kcal/mol)	0.490	0.280	0.390
Steric zipper^b^ hydrophobicity (kcal/mol)	0.675	0.218	0.664
Zag propensity log K(s^−1^)	−4.3	−4.5	−3.2
Aromatic (X) and positive polar (P) amino acids position into the peptide sequence			
Globular structure formation	Yes	Yes	Yes
Fibril formation	Yes	No	Yes
Cytotoxicity	Medium	Poor	High

^a^amino acids: positive (*blue*), negative (*red*), polar uncharged (*green*), aromatic (*yellow*)

^b^The steric zipper is underlined

### Circular dichroism

**Fig. 2:**
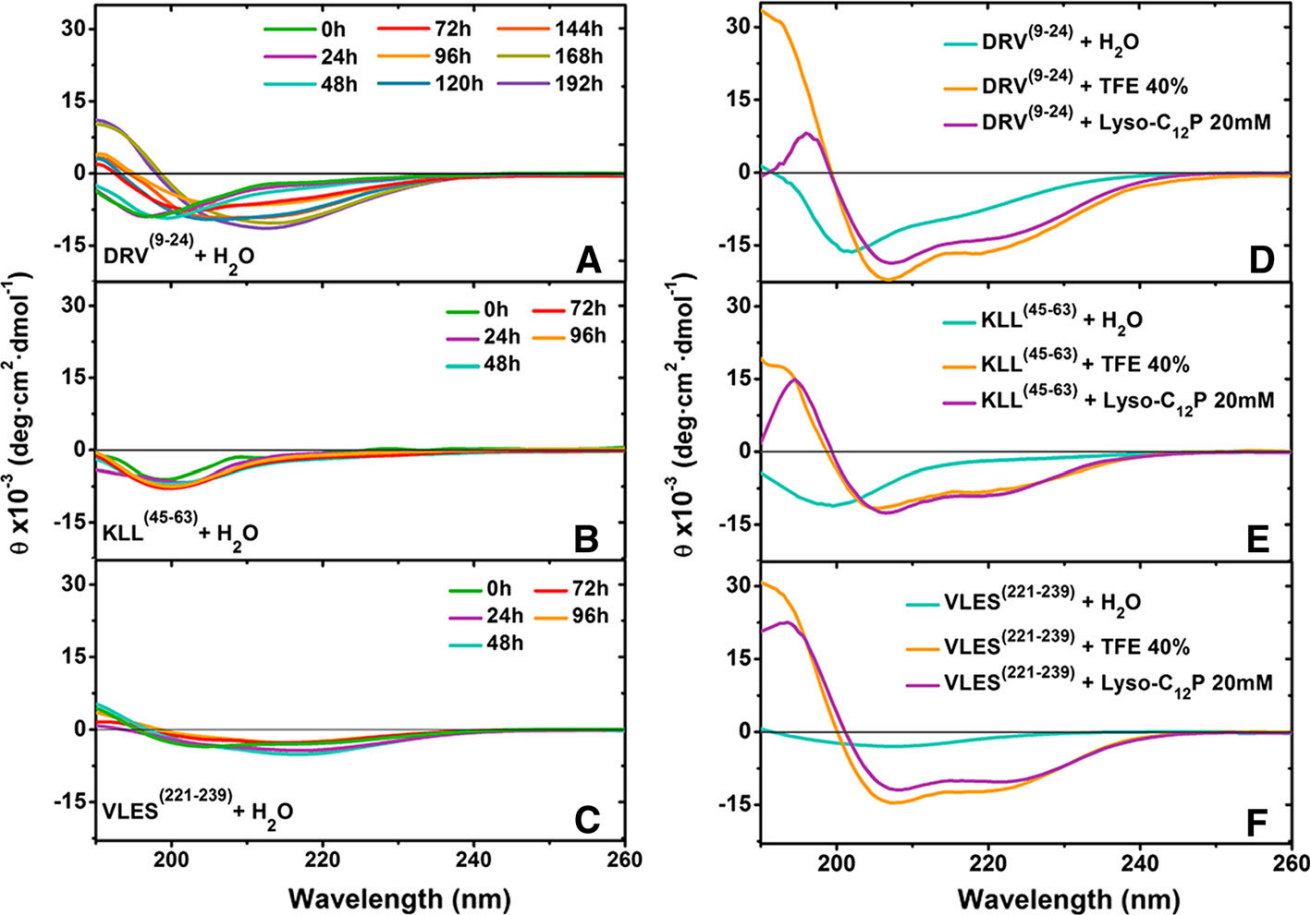
Far-UV CD spectra of apoA-I derived peptides. **a–c** Spectra of peptide DRV^(9–24)^, KLL^(45–63)^, and VLES^(221–239)^ incubated for various times in water. **d–f** CD spectra of peptides DRV^(9–24)^, KLL^(45–63)^, and VLES^(221–239)^ incubated for 24 h in 40 % TFE and 20 mM Lyso-C_12_PC

Peptides DRV^(9–24)^, KLL^(45–63)^, and VLES^(221–239)^ placed in water were monitored by far-UV CD at 24 h intervals (Fig. 2a–c) in the presence of 20 mM Lyso-C_12_PC (cmc = 0.9 mM) or 40 % TFE (Fig. 2d–f). The percentages for the different secondary structural modules for these peptides were calculated employing the CDNN program (Table 2). CD spectra of peptides dissolved in water presented a single minimum around 200 nm at the 0 timepoint, indicating primarily the presence of disordered structures. The CD spectra deconvolution of DRV^(9–24)^, KLL^(45–63)^, and VLES^(221–239)^ in water displayed a mixture of α-helix, β-sheet, and disordered structures (Table 2). In the presence of Lyso-C_12_PC and TFE, the three peptides showed a clear minimum at 208 and 222 nm and a maximum at 195 nm, characteristic of an α-helix (Fig. 2d–f). Lyso-C_12_PC strongly promoted the recovery of secondary structure from a disordered conformation with all peptides tested. Optimal recoveries occurred with peptide VLES^(221–239)^, whereas the weakest one was shown by peptide KLL^(45–63)^ (Fig. 2d–f).

**Table 2:** Secondary structure content of apoA-I derived peptides in water and in the presence of hydrophobic molecules

Sample	Incubation time (h)	PEPTIDES								
		DRV^(9–24)^			KLL^(45–63)^			VLES^(221–239)^		
		% *α*	% *β*	% rc	% *α*	% *β*	% rc	% *α*	% *β*	% rc
H_2_O	0	6.6	35.3	38.4	4.5	38.2	35.4	8.5	46.0	28.6
	24	7.0	34.1	38.2	5.0	36.9	35.3	10.5	47.8	27.0
	48	7.3	32.3	38.4	5.1	36.4	35.2	11.1	45.2	27.7
	72	9.7	27.7	37.2	4.2	39.5	35.2	10.3	50.2	26.2
	96	11.2	26.9	36.4	4.0	41.2	35.2	10.3	46.0	27.6
	120	12.7	22.0	36.8						
	144	13.1	22.0	36.5						
	168	16.8	19.8	34.7						
	192	17.6	18.4	34.5						
TFE 40 %	24	47.2	0.6	26.7	26.3	19.4	28.0	39.5	2.7	25.0
Lyso-C_12_PC 20 mM	24	26.8	3.9	46.3	24.2	13.4	28.0	34.0	5.9	26.0

TFE (2,2,2-Trifluoroethanol), Lyso-C_12_PC (1-lauroyl-2-hydroxy-*sn*-glycero-3-phosphocholine). *α* (α-helix), *β* (β-sheet), rc (random coil). Secondary structure percentages were estimated using the circular dichroism neural network (CDNN) based software

### Peptide aggregates observed by TEM and AFM

**Fig. 3:**
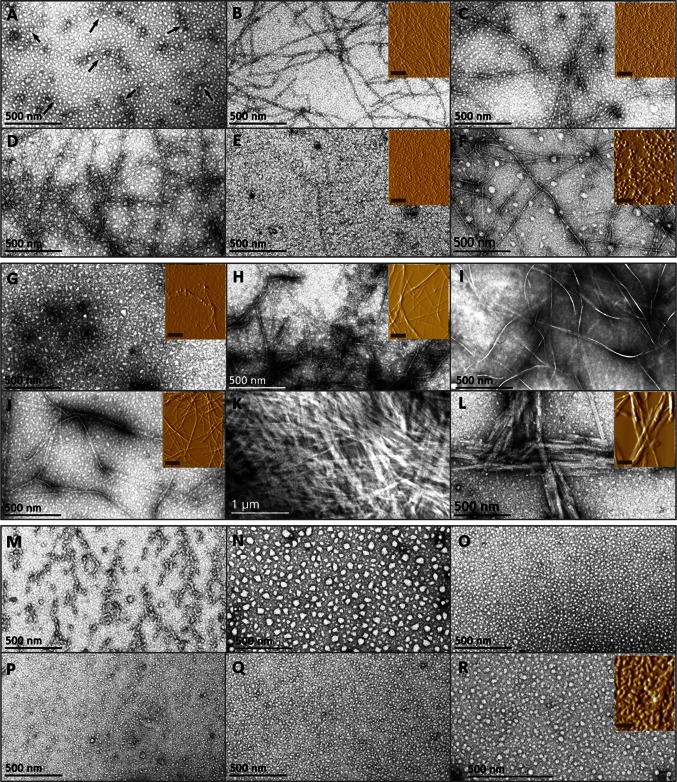
TEM and AFM micrographs of peptides DRV^(9–24)^, KLL^(45–63)^, and VLES^(221–239)^. Evolution of peptide aggregation in water was evaluated by electron and atomic force microscopies on peptides VLES^(221–239)^ (*A–F*), DRV^(9–24)^ (*G–L*), and KLL^(45–63)^ (*M–R*). Incubation times correspond to: 0 h (*A, G, M*), 24 h (*B, H, N*), 48 h (*C, I, O*), 72 h (*D, J, P*), 92 h (*E, K, Q*), and 120 days (*F, L, R*). *Arrows* (*A*) show protofibrillar structures. Insets correspond to AFM images of samples used in CD experiments and the scale correspond to 1 µm

Since an increase in β-sheet structures suggests the potential formation of amyloid-like fibrils, peptide samples were analyzed by TEM (Fig. 3) and AFM (Fig. 3 insets). This figure shows the different configurations that peptides VLES^(221–239)^ (Fig. 3a–f), DRV^(9–24)^ (Fig. 3g–l) and KLL^(45–63)^ (Fig. 3 m–r) display over time. Peptide VLES^(221–239)^ at 0 h revealed protofibrillar structures (arrows, Fig. 3a) that longitudinally grow after 24 h incubation (Fig. 3b). After 48, 72, and 96 h incubation (Fig. 3c–e), fibrillar or globular structures did not show any modification. However, after 120 days incubation, peptide VLES^(221–239)^ shows the formation of globular structures that significantly increased in size (Fig. 3f). Peptide DRV^(9–24)^ displayed a mixture of oligomers and protofibrillar intermediaries at the start of the experiment (Fig. 3g), whereas at 24 h incubation, we observed long fibrillar structures (Fig. 3h). After 48 and 72 h incubation, lateral aggregation of fibrils generated wider structures (Fig. 3i,j). At 96 h incubation, thick bundles of fibrillar structures were found with no additional changes in size seen after 120 days incubation (Fig. 3k,l). In contrast, no fibrillar aggregates were seen during the incubation of KLL^(45–63)^ peptide over time (Fig. 3m–r).

### MTT cell viability assay and optical microscopy

**Fig. 4:**
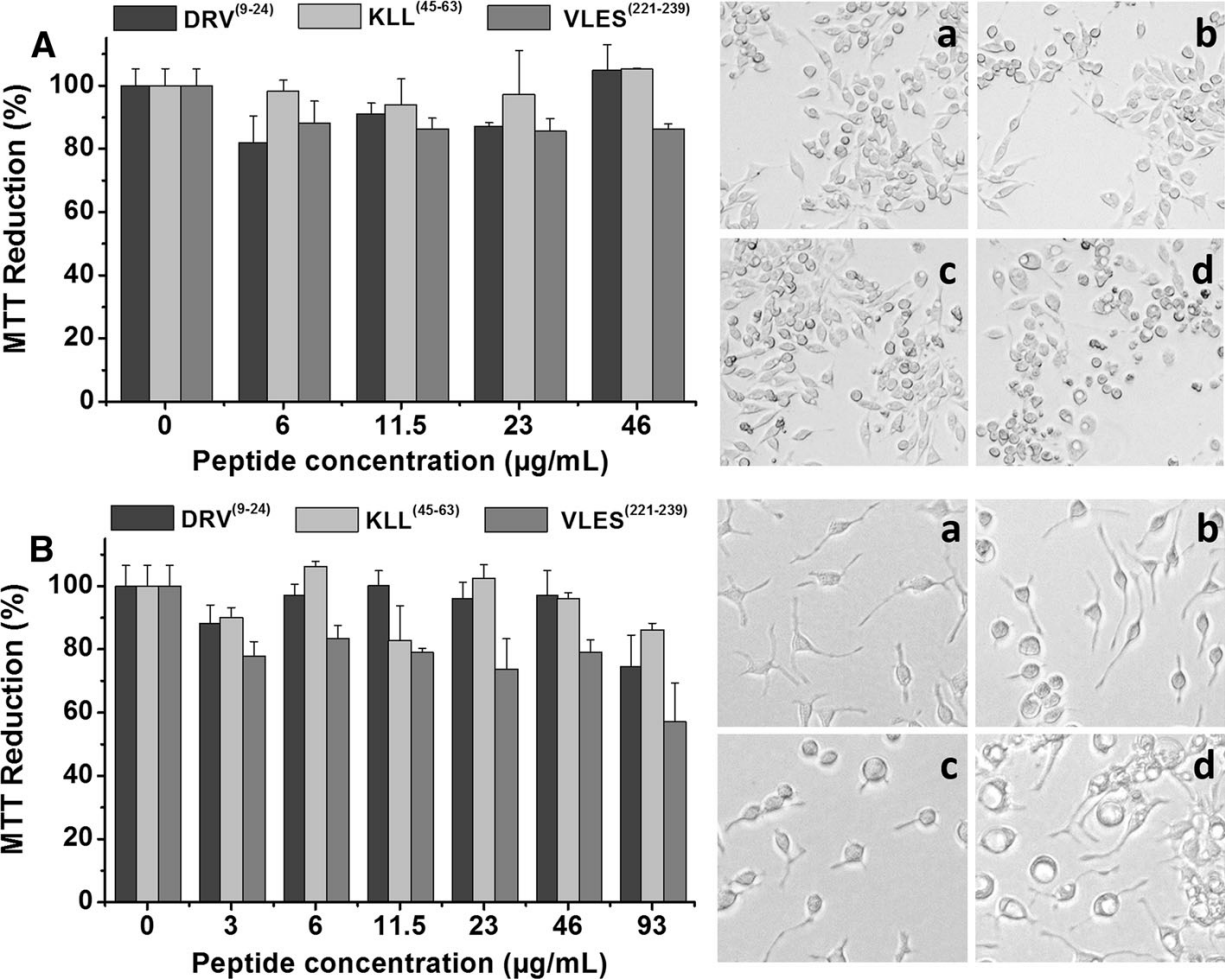
Cytotoxic effects of apoA-I derived peptides. MTT reductions experiments performed with macrophage (**A)** and microglial cells (**B**). **Aa-d** Optical microscopy images of macrophages treated for 24 h with peptides previously incubated at 4 °C in water for 120 days (46 µg/mL). Ba-d) Optical microscopy images of microglial cells treated for 24 h with peptides previously incubated at 4 °C in water for 120 days (93 µg/mL). **a** Control without peptides, **b** DRV^(9–24)^, **c** KLL^(45–63)^, and **d** VLES^(221–239)^

As previously reported by us employing the β-amyloid peptide, cytotoxic effects of aggregates formed by DRV^(9–24)^, KLL^(45–63)^ and VLES^(221–239)^ were evaluated on macrophage and microglial cells [27], following the reduction of MTT as an indirect indicator of cellular oxidative stress [28]. DRV^(9–24)^, KLL^(45–63)^ and VLES^(221–239)^ peptides were incubated for 120 days prior to viability testing. When macrophages were exposed to increasing concentrations of the three tested peptides, cell viability was only slightly decreased in the case of VLES^(221–239)^, with no dependence on peptide concentration (Fig. 4A). For the viability experiment performed with microglial cells, the peptide concentration range was expanded to 93 µg/mL and a clear loss of cell viability was observed (Fig. 4B). Optical microscopy images shown in Fig. 4Ad and 4Bd depict clear morphological changes in both cell types treated with peptide VLES^(221–239)^. At the highest concentration tested, vacuolization was registered in macrophages employing 46 µg VLES/mL and with 93 µg VLES/mL for microglial cells. Modest cytotoxic effects were observed with both peptides DRV^(9–24)^ (Fig. 4Ab, Bb) and KLL^(45–63)^ (Fig. 4Ac, Bc). From these series of cell viability experiments in correlation to peptide aggregates shown by TEM and AFM, it becomes clear that VLES^(221–239)^ with the presence of small protofilaments associated to oligomers corresponds to the most toxic form of the peptide.

## Discussion

In 1950, Karush [29] proposed that protein–ligand interactions stabilized the best-fitting members within an assembly of structures in equilibrium. Since then, numerous studies have demonstrated that disorder and flexibility in protein structure are important features in the understanding of protein function. In the case of apolipoproteins such as apoA-I, apoC-II, and apoE, it has been found that they correspond to intrinsically disordered proteins. As a consequence of their structural flexibility, the X-ray crystal structure of the C-terminal apoA-I has shown its propensity to destabilization as well as to be able to adopt different conformations when associated to lipids or in a lipid-free state [30, 31]. The crystal structure of lipid-free apoA-I demonstrated that although it contains a largely helical four-segment bundle [31], when in solution, this segment adopts a molten globule conformation with its N- terminal domain completely disordered [7].

When apolipoproteins are exposed to air/water and lipid/water interfaces, evident disorder-to-order conformational transitions take place that might have an important impact upon HDL function [32–34]. In this sense, we have previously shown that conformational transitions observed with a series of apoA-I derived peptides stabilize and improve the enzymatic activity of LCAT [19].

In order to predict which segments of apoA-I could present the greater propensity to develop disorder-to-order transitions, using the PONDR-FIT server, we have studied three segments that bind to lipids and acquire a helical conformation that might contribute to the stabilization of lipid-protein interaction. Within each of the disordered N- and C-terminal domains, small segments of hydrophobic β-sheet structure are exposed and, therefore, proposed to interact with lipids [7, 20]. Our analysis using the HCA server revealed three highly hydrophobic clusters within the helical structure of apoA-I, being the longest one found at the C-terminal domain. This result supports our previous reports and also suggests that lipid-free apoA-I first binds membrane lipids or surface lipids of lipoproteins through the C-terminal fragment. The presence of nonpolar core residues in the protein may be related with this phenomenon, which does not occur when proteins have an inadequate number of hydrophobic residues.

Peptides studied in this investigation have the particular feature of having a good balance between charge and hydrophobicity that allows them to stay “suspended” as globular structures and due to hydrophobic interactions bound together with the potential to generate fibrillar structures. While peptide DRV^(9–24)^ having a charge of −1 presents the ability to easily generate amyloid-like fibers as previously reported by us [27], peptide VLES^(221–239)^ with no charge at physiological pH, only form thin protofibrils. Peptide KLL^(45–63)^ also presenting a net charge of 0 at physiological pH, shows a low efficiency for intermolecular interactions and, therefore, a low propensity for the generation of fibrils.

Peptides and proteins capable of generating amyloid fibers present common hydrophobic structural blocks called “steric zippers” [35]. Interestingly, DRV^(9–24)^ in addition to presenting the highest µH, also contains a “steric zipper” with the highest average hydrophobicity value when compared to peptides KLL^(45–63)^ and VLES^(221–239)^. Although peptide KLL^(45–63)^ is the most polar of the three peptides tested, it also contains a “steric zipper” showing weaker hydrophobic characteristics than those present in peptides DRV^(9–24)^ and VLES^(221–239)^. This characteristic indicates the absence of an adequate nucleation center that might self-assemble and, therefore, the difficulty to generate fibrillar structures. CD spectra deconvolution for peptides DRV^(9–24)^ and VLES^(221–239)^ suspended in water, showed over time a moderate increase in α-helix content despite microscopy evidence of fibril formation (Table 2). CD spectra deconvolution for peptide KLL^(45–63)^ suggests that the formation of β-sheet structures alone is insufficient to induce protein aggregation and fibril formation. On the other hand, the use of the software Zyggregator predicted that propensity for amyloid-like fibril formation was similar for peptides DRV^(9–24)^, KLL^(45–63)^, and VLES^(221–239)^ (Fig. 2b).

**Fig. 5:**
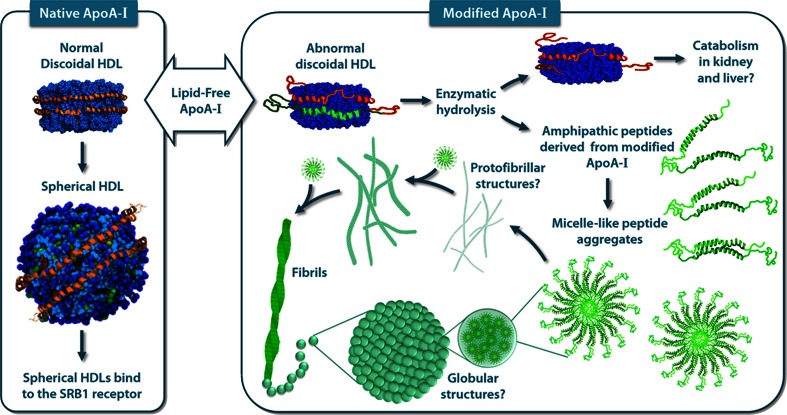
ApoA-I aggregation properties. Lipid-poor apoA-I interacts with the ABCA1 receptor and produce discoidal structures following a process still under active investigation. Discoidal particles are transformed into spherical HDLs by the action of the LCAT enzyme [39]. Only the spherical forms of HDL can interact with the SRB1 receptor. Modified apoA-I can not interact properly with the ABCA1 receptor forming smaller abnormal discoidal HDLs [40]. A direct consequence of this condition is the exposure of highly unstructured apoA-I segments prone to enzymatic hydrolysis [41]. Peptides released by proteolysis might form amyloidogenic structures that can be organized first as micelle-like peptides that can evolve to form globular or protofibrillar structures depending on their residue composition [42, 47]. Modified from [33]

We recognize three elemental features in peptides DRV^(9–24)^ and VLES^(221–239)^ that lead to protein aggregation. In the first place, peptides present a steric zipper characteristic of amyloidogenic proteins in the form of tandems of hydrophobic and nonpolar amino acids (Table 1). Second, the presence of aromatic amino acids, in particular phenylalanine, is related with self-assembly of amyloid fibrils. Finally, we observed a size uniformity of the steric zipper and adjacent regions, a property related to protein–protein interactions that drive amyloid-like formation. The existence of unstructured segments at the N- and C-terminal domains of apoA-I makes the protein susceptible to enzymatic hydrolysis [36], phenomenon that increases the probability for the generation of highly hydrophobic autoimmune structures that in turn might induce an inflammatory response [33, 37, 38] promoting the generation of nucleation centers important in the formation of amyloid-like fibrils (Fig. 5) [39–42]. In addition, under proteolytic conditions, it has been observed that apoA-I/HDL releases peptides from its terminal domains showing the same properties as when they are in a lipid medium [43, 44]. During our viability assays, peptide VLES^(221–239)^ and to a lesser extent DRV^(9–24)^, the two peptides that generate fibers, have the ability to promote cytotoxicity. VLES^(221–239)^ also presents the property to alter the cell membrane as observed by optical microscopy (Fig. 4Bd). Conducting an analysis of peptide sequence motifs capable of interacting with membranes or phospholipidic surfaces [45, 46], it is observed that peptide DRV^(9–24)^ presents amino acids with a net positive charge (lysine and arginine) and aromatic amino acids. Interestingly, peptide KLL^(45–63)^ showing a lysine residue at the N- terminal, two aromatic amino acids, and several non-polar residues resembling the phospholipid polar head might promote the formation of “peptide micelles” [47]. It has been established that electrostatic interactions between positive residues located at the protein and the presence of negatively charged phospholipids, are the dominant forces that promote adsorption of the protein onto the membrane surface [48]. On the other hand, nonpolar interactions between hydrophobic segments of the protein and the presence of lipid hydrophobic chains, lead to the insertion of the protein into the membrane interface [45]. In our hands, incubation of cells in a medium containing VLES^(221–239)^ showing an α-helical structure when associated to lipid molecules, seems to be the conformation that mostly damage cells [40, 49]. From the images seen with TEM and AFM, it can be concluded that the presence of VLES^(221–239)^ in the form of small protofilaments and oligomers corresponds to structural forms associated to a high degree of cytotoxicity.

Based on the conformational transitions and cytotoxicity associated to the apoA-I derived peptides used in this work, we suggest that transitions leading to an α-helix formation in this protein at the hydrophilic/hydrophobic interface of membranes can be considered a key feature to explain cell toxicity.

Our study puts into perspective the fact that highly hydrophobic segments of apoA-I present the ability to develop secondary structure disorder-to-order transitions depending on the molecules to which it is associated. The association of these highly hydrophobic segments to specific types of lipid molecules could shift the equilibrium toward the consolidation of α-helical segments that would apparently warranty the normal function of the protein. In contrast, if these segments follow protein–protein interactions or are kept in highly hydrophilic environments, the possibility for the generation of localized pro-aggregation structures might disrupt the normal function of apoA-I.

The dynamic structure exhibited by apoA-I basically supported by intrinsically disordered exposed segments that undergo disorder-to-order and order-to-disorder conformational transitions might also explain the exchangeability properties shown by this family of apolipoproteins. When the protein is located in a highly hydrophilic media with its lateral segments exposed, these segments mostly show a disordered conformation and the permanence of the protein in plasma maintained. Nevertheless, when these segments start to get associated to lipid, there is a shift toward organized secondary conformational structures mostly α-helical structures that in a continuum tend to change the equilibrium, toward the formation and consolidation of lipid loaded particles that eventually give rise to the generation of HDL.

For many years, protein function had resided in the fact that well-ordered structures mostly through rigid tridimensional blocks were fundamental for understanding the way proteins work. Nevertheless, nowadays this concept has been surpassed when recognition has been made to phylogenetically advanced organisms that develop function through and important number of intrinsically disordered proteins and the concept of structural disorder, as a new form of secondary structure of proteins conceived. In this sense, an important number of diseases that in the past had been difficult to understand, during the present days they start to find an explanation in the anomalous folding of proteins [50]. Without a doubt, we can say that, in the near future many diseases with poorly understood origins not only will find a molecular explanation based on this phenomenon, but also in the way intrinsically disordered regions of proteins are modulated.
